# The effect of radiolabeled nanostructured lipid carrier systems containing imatinib mesylate on NIH-3T3 and CRL-1739 cells

**DOI:** 10.1080/10717544.2020.1841337

**Published:** 2020-12-02

**Authors:** Evren Atlihan Gundogdu, Emine Selin Demir, Meliha Ekinci, Emre Ozgenc, Derya Ilem Ozdemir, Zeynep Senyigit, Makbule Asikoglu

**Affiliations:** Department of Radiopharmacy, Faculty of Pharmacy, Ege University, Izmir, Turkey

**Keywords:** Nanostructured lipid carrier systems, cell culture, characterization, radiolabeling, Technetium-99m

## Abstract

The aim of current study is to develop new nanostructured lipid carrier systems (NLCSs) containing imatinib mesylate (IMT) and evaluate their targeting efficiency on NIH-3T3 as fibroblast cells and CRL-1739 as gastric adenocarcinoma cells with radiolabeled formulations. Three formulations (F1-IMT, F2-IMT and F3-IMT) were prepared and radiolabeled with 1 mCi/0.1 mL of [^99m^Tc]Tc. The effect of reducing and antioxidant agents on radiolabeling process was evaluated and radiochemical purity of formulations was performed by radio thin-layer radiochromatography (RTLC). The results demonstrated that the radiochemical purity was found to be above 90% for [^99m^Tc]Tc-F1-IMT and [^99m^Tc]Tc-F2-IMT, while radiochemical purity of [^99m^Tc]Tc-F3-IMT was found to be 85.61 ± 2.24%. Also, [^99m^Tc]Tc-F1-IMT and [^99m^Tc]Tc-F2-IMT have better stability in cell medium and saline than [^99m^Tc]Tc-F3-IMT. Targeting efficiency of [^99m^Tc]Tc-F1-IMT and [^99m^Tc]Tc-F2-IMT comparatively evaluated by cell binding studies with [^99m^Tc]NaTcO4 on NIH-3T3 and CRL-1739 cells. The cell binding capacity and targeting/non-targeting cell uptake ratio of these two formulations was found to be higher than [^99m^Tc]NaTcO4 in CRL-1739. It is thought that the knowledge achieved in this study would contribute to using [^99m^Tc]Tc-F1-IMT and [^99m^Tc]Tc F2-IMT as an diagnosis and treatment agents.

## Introduction

Imatinib mesylate (IMT) is tyrosine kinase enzyme inhibitor and extensively used for the treatment of chronic myelogenous leukemia and gastrointestinal stromal tumors (GISTs). Furthermore, many patients with IMT therapy have dose-dependent adverse effects including cardiac, pulmonary, and hepatic toxicities (Blay et al., 2012). These factors decrease IMT treatment efficiency. Therefore, convenient drug delivery system for IMT administration may play an important role for new therapeutic approach to target GISTs that increments the concentration at the desired cell, while decreases the toxicity for other cells.

Nanostructured lipid carrier systems (NLCSs) are part of drug delivery systems and comprised of solid and liquid lipids. They have many advantages such as high drug loading capacity (LC), easy to prepare, biocompatible facility, low cost, good stability, long shelf-life, and ease of storage (Khosa et al., 2018). Also, their usage with convenient radionuclides is an attracting major attention in nuclear medicine. Technetium-99m ([^99m^Tc]Tc) is commonly preferred radionuclide for radiolabeling of drug delivery systems since it has ideal properties such as short half-life (6 h), low cost, availability through an inexpensive ^99^Mo/^99m^Tc generator, varied chemistry, obtains a high activity to be given to the patient without an excessive radiation dose, readily detectable gamma emission (Ucar et al., 2017; Fernandes et al., 2018).

The aim of this work was to develop new NLCSs containing IMT and evaluate their targeting efficiency on NIH-3T3 as fibroblast cells and CRL-1739 as gastric adenocarcinoma cells with radiolabeled formulations. First, NLCSs containing IMT were developed. They were evaluated in terms of particle size, zeta potential, polydispersity index (PDI), entrapment efficiency (EE), LC, stability, and cytotoxicity studies. The developed formulations were radiolabeled with [^99m^Tc]Tc. The effects of reducing and antioxidant agents on radiochemical purity were investigated. The *in vitro* stability of radiolabeled NLCSs containing IMT ([^99m^Tc]Tc-NLCS-IMT formulations) was determined in saline and cell medium. Finally, cell binding studies of technetium pertechnatate ([^99m^Tc]NaTcO_4_), and [^99m^Tc]Tc-NLCS-IMT formulations were comparatively performed by using NIH-3T3 and CRL-1739 cells to evaluate targeting efficiency of formulations.

## Materials and methods

### Materials

Gelucire pellet and lipoid derivatives were obtained from Gattefosse Sas (Saint-Priest, France) and LIPOID GmbH (Ludwigshafen, Germany), respectively. Imatinib (Gleevec^®^) was obtained from Novartis (Basel, Switzerland). NIH-3T3 and CRL-173 cells were purchased from ATCC (Manassas, VA). [^99m^Tc]NaTcO_4_ was obtained from Department of Nuclear Medicine, Ege University (İzmir, Turkey).

### Preparation of NLCS containing IMT

The NLCS were prepared by the emulsification-sonication methods (Müller et al., 2000; Pardeike et al., 2009). Briefly, the lipids comprised of oleic acid and compritol, oleic acid and gelucire derivatives (gelucire 48/16 and gelucire 43/01 pellets) were heated in a water bath (70–80 °C). Span80, Tween 80, and lipoid derivatives were used as surfactants. One milligram of IMT was dissolved in lipid phase and an aqueous solution containing surfactants heated to the same temperature as the lipid phase. The aqueous and oily phases were mixed under high-speed stirring (10,000 rpm) for 10 min by using an Ultra-Turrax blender. The sonication was applied to the obtained emulsion at 500 W and 20 kHz in changing 20 s cycles for 15 min with Vibracell tip sonicator and then immediately cooled to room temperature. Three formulations (F1-IMT, F2-IMT, and F3-IMT) were obtained at the end of preparation.

### Characterization and stability studies of NLCS containing IMT

The particle size and PDI of formulations (F1-IMT, F2-IMT, and F3-IMT) were evaluated with Malvern Zetasizer (Malvern Nano ZS 90, Worcestershire, UK) at room temperature. The zeta potential of formulations was also measured at 40 V/cm using a DTS 1060 C zeta cuvette at room temperature (*n* = 6).

The EE and LC of F1-IMT, F2-IMT, and F3-IMT were performed by using dialysis bag that has 8000–12,000 Da of molecular weight. The formulations were implemented in dialysis bags. The samples were ultracentrifuged in 5000 rev/min at room temperature and filtered by using cellulose nitrate membrane. The amount of IMT was measured by UPLC. The EE and LC were calculated according to the following equations (Wang et al., 2013; Kanwar et al., 2018):

$$\text{EE}\;\left(\mu g\right)\;=\;\left(\left(W_{\text{total}\;\text{drug}}-\;W_{\text{free}\;\text{drug}}\right)/W_{\text{total}\;\text{drug}}\right)\;\times \;1$$

$$\text{LC}\;\left(\%\right)\;=\;\left(\left(W_{\text{total}\;\text{drug}}-\;W_{\text{free}\;\text{drug}}\right)/W_{\text{total}\;\text{formulation}}\right)\;\times \;1$$

$$W_{\text{total}\;\text{drug}}=\text{the}\;\text{total}\;\text{amount}\;\text{of}\;\text{drug}$$

$$W_{\text{free}\;\text{drug}}=\text{the}\;\text{amount}\;\text{of}\;\text{drug}\;\text{into}\;\text{the}\;\text{supernatant}$$

$$W_{\text{total}\;\text{formulation}}=\text{the}\;\text{amount}\;\text{of}\;\text{formulation}$$

The stability of F1-IMT, F2-IMT, and F3-IMT was observed during 12 months at 5 ± 3 °C, 25 ± 5 °C, and 60 ± 5% relative humidity (RH), 40 ± 5 °C and 75 ± 5% (RH). The particle size, PDI, zeta potential, EE, LC of formulations and visual inspection of formulations appearance were evaluated. Analysis of variance (ANOVA, 95% confidence level) was used to compare the initial and subsequent values.

### Cell culture studies

NIH-3T3 and CRL-1739 were used for *in vitro* cytotoxicity and cell binding studies. The cells were grown in Dulbecco’s modified Eagle medium (DMEM) supplemented with 10% fetal bovine serum in a humidified atmosphere with 5% CO_2_ at 37 °C. The cells were cultured in 25 cm^2^ flasks until they reached 80–90% confluence. They were seeded at a density of 1 × 10^6^ cells/well and 2 × 10^6^ cells/well for *in vitro* cytotoxicity and cell binding studies, respectively.

### *In vitro* cytotoxicity studies

The *in vitro* cytotoxicity of F1-IMT, F2-IMT, F3-IMT, and IMT solution (in cell medium) with different concentrations (0.05 mg/mL, 0.1 mg/mL, 0.2 mg/mL, 0.4 mg/mL, 0.5 mg/mL, 1 mg/mL, and 2 mg/mL) was performed by using 3-[4,5-dimethylthiazole-2-yl]-2,5-diphenyltetrazolium bromide (MTT). They were incubated with NIH-3T3 and CRL-1739 cells during 24 and 48 h. After incubation times, 100 μg/mL of MTT in sterile phosphate buffer (PBS) was put into each well. The culture medium was removed and 200 μL of dimethysulfoxide was added to obtain dissolution of the MTT formazan crystals. The absorbance read at 570 nm in microplate reader. ‘GraphPad Prism’ and ‘One-site total binding’ algorithm programs were used to calculate 50% inhibition of viability (IC_50_) (Bondì et al., 2007; Liu et al., 2011).

### Preparation of [^99m^Tc]Tc-NLCS-IMT formulations

The radiolabeled formulations were called [^99m^Tc]Tc-NLCS-IMT formulations (F1-IMT, F2-IMT, and F3-IMT). They were prepared with 1 mCi/0.1 mL of [^99m^Tc]Tc. The effect of reducing and antioxidant agents was investigated to find the optimum radiolabeling conditions. Radio thin‐layer radiochromatography (RTLC) was used to determine the radiochemical purity.

Five groups (*n* = 6) were obtained to investigate the effect of reducing agent. Ten milligrams of F1-IMT, F2-IMT, and F3-IMT was dissolved in 1 mL of saline. Reduction of ^99m^Tc was performed with different amount of stannous chloride in 0.01 N HCl (10, 50, 250, 500, and 1000 µg). Stannous chloride solution was added to the formulations, under an atmosphere of bubbling nitrogen. Radiolabeling was made with 1 mCi/0.1 mL of ^99m^Tc in saline. The radiolabeled formulations were mixed for one minute in vortex mixer and incubated for 15 minutes at room temperature. The 50 µL of samples were taken from each formulations during 6 h and radiochemical purity of samples was analyzed with RTLC. The contents of the formulations to examine the effect of reducing agent on radiolabeling process are shown in Table 1.

**Table 1.** The formulations to examine the effect of stannous chloride amounts on radiolabeling process.

Group numbers	Formulations	Stannous chloride (µg/mL)	Tc-99m (mCi)
1	F1-IMT	10	1
	F2-IMT	10	1
	F3-IMT	10	1
2	F1-IMT	50	1
	F2-IMT	50	1
	F3-IMT	50	1
3	F1-IMT	250	1
	F2-IMT	250	1
	F3-IMT	250	1
4	F1-IMT	500	1
	F2-IMT	500	1
	F3-IMT	500	1
5	F1-IMT	1000	1
	F2-IMT	1000	1
	F3-IMT	1000	1

Ten main groups (*n* = 6) were obtained to investigate the effect of antioxidant agent. Ten milligrams of F1-IMT, F2-IMT, and F3-IMT were dissolved in 1 mL of saline. While stannous chloride and 0.1 mg ascorbic acid were added to the formulations in the subgroups, stannous chloride and 0.5 mg ascorbic acid were put into the formulations in the other subgroups (Table 2). Radiolabeling was made with 1 mCi/0.1 mL of ^99m^Tc in saline. The radiolabeled formulations were mixed for one minute in a vortex mixer and incubated for 15 minutes at room temperature. The 50 µL of samples were taken from each formulations during 6 h and radiochemical purity of samples was analyzed with RTLC.

**Table 2.** The formulations to examine the effect of ascorbic acid amounts on radiolabeling process.

Group numbers	Formulations	Stannous chloride (µg/mL)	Ascorbic acid (mg)	Tc-99m (mCi)
1	F1-IMT	10	0.1	1
	F2-IMT	10	0.1	1
	F3-IMT	10	0.1	1
2	F1-IMT	10	0.5	1
	F2-IMT	10	0.5	1
	F3-IMT	10	0.5	1
3	F1-IMT	50	0.1	1
	F2-IMT	50	0.1	1
	F3-IMT	50	0.1	1
4	F1-IMT	50	0.5	1
	F2-IMT	50	0.5	1
	F3-IMT	50	0.5	1
5	F1-IMT	250	0.1	1
	F2-IMT	250	0.1	1
	F3-IMT	250	0.1	1
6	F1-IMT	250	0.5	1
	F2-IMT	250	0.5	1
	F3-IMT	250	0.5	1
7	F1-IMT	500	0.1	1
	F2-IMT	500	0.1	1
	F3-IMT	500	0.1	1
8	F1-IMT	500	0.5	1
	F2-IMT	500	0.5	1
	F3-IMT	500	0.5	1
9	F1-IMT	1000	0.1	1
	F2-IMT	1000	0.1	1
	F3-IMT	1000	0.1	1
10	F1-IMT	1000	0.5	1
	F2-IMT	1000	0.5	1
	F3-IMT	1000	0.5	1

### Evaluation of radiochemical purity for [^99m^Tc]Tc-NLCS-IMT formulations

The radiochemical purity of [^99m^Tc]Tc-NLCS-IMT formulations was assessed by RTLC (Bioscan AR 2000, Bioscan, Inc., Washington, DC) up to 6 h. Instant thin layer chromatography-silica gel coated fiber sheets (ITLC-SG) was used as stationary phase. The free [^99m^Tc]Tc and reduced/hydrolyzed (R/H) [^99m^Tc]Tc were determined by using saline and pyridine:acetic acid:water (3:5:1.4)) as mobile phases. The radiochemical purity percentage (RP%) was calculated from the following equation (İlem-Özdemir et al., 2016):

$$\text{RP}\%=1\;–\;\left({\text{free}\;}^{99m}\text{Tc}\;\left(\%\right)\;+{\;R/H}^{99m}\text{Tc}\;\left(\%\right)\right)$$

### *In vitro* stability of [^99m^Tc]Tc-NLCS-IMT formulations

[^99m^Tc]Tc-NLCS-IMT formulations were prepared by using the most appropriate stannous chloride and ascorbic acid amounts. Two hundred microliters [^99m^Tc]Tc-NLCS-IMT formulations were incubated with 800 µL saline and cell medium at 37 °C. The samples were assayed up to 6 h by using RTLC.

### Cell binding studies with [^99m^Tc]Tc-NLCS-IMT formulations

The cell binding studies were performed with NIH-3T3 and CRL-1739 cells to investigate targeting efficiency of formulations. The cells were incubated with 1 mCi of [^99m^Tc]NaTcO_4_, [^99m^Tc]Tc-F1-IMT, and [^99m^Tc]Tc-F2-IMT for 30, 60, and 120 min at 37 °C. The cell culture medium was collected and cells were treated with 0.5 mL of trypsin to remove the cells. They were consecutively washed with 1 mL of cell medium and PBS. The activities in the tubes containing sediment cells and cell culture medium were both counted by a gamma counter (Sesa Uniscaller). The cell binding ratio of radiolabeled formulations was calculated from the following equation:

Cell binding ratio of radiolabeled formulations (%) = 1 × (radioactivity of cells/total radioactivity)

The targeting/non-targeting cell uptake ratio for all formulations was also found by dividing the cell binding ratio of radiolabeled formulations for CRL-1739 to cell binding ratio of radiolabeled formulations for NIH-3T3 (Elitez et al., 2018).

### Statistical analysis

The means and standard deviations of results were calculated on Microsoft Excel (Redmond, WA). Statistical analysis was performed by using Oneway Anova program. Differences at the 95% confidence level (*p*<.05) were considered significant. Experiments were performed in triplicate. Results are reported as mean ± standard error.

## Results

### Preparation of NLCS containing IMT

Some formulations having different ratio of vehicles have been assayed. Some of them were excluded because of foam formation and phase separation during preparation process (Table 3). In addition, the ideal formulations were determined with particle size, PDI, and zeta potential values. Owing to the higher solubilizing capacity of oleic acid for lipophilic drugs, it has been selected as the liquid lipid (Negi et al., 2014). A liquid lipid (oleic acid) was utilized with the solid lipid (gelucire derivatives and compritol 888) into the lipid phase to improve the drug phase separation movement. The polysorbate class of amphiphilics which can lead to emulsification of the lipidic mixture and their combination with gelucire derivatives and compritol 888 is caused by formation of nanostructures and providing stabilization of NLCS. Hydrophilic solvents such as water, ethanol, propanol, butanol, pentanol, and hexanol have critical role in the formation of NLCS. They provide rapid distribution of lipid phase into the aqueous phase (Qu et al., 2016). Especially, ethanol and water have available properties such as low toxicity, facilitation of forming NLCSs for pharmaceutical administration with small particle size (Schubert et al., 2011; Chin et al., 2014). Herein, water and water:acetone:ethanol mixture were used. The emulsification-sonication method has advantages such as ease of production process, short process time, homogeneous particle size, particle size reduction and obtaining stable formulation. Furthermore, it is available for the scale-up production (Hu et al., 2016; Amasya et al., 2019). In this study, stable NLCSs were developed by these techniques. The desired particle size was achieved by 500 W and 20 kHz, 10 min at 10,000 rpm. As a result, three formulations (F1-IMT, F2-IMT, and F3-IMT) were obtained with different ratios and contents and prepared with different stirring times and same sonication condition (Table 3).

**Table 3.** The contents and preparation conditions of F1-IMT, F2-IMT, F3-IMT, and other formulations.

Formulations	Gelucire 48/16 pellets (%)	Oleic acid (%)	Span 80 (%)	Lipoid S 100 (%)	Water:acetone:ethanol (5:2.5:2.5 v/v) %	Water (%)	IMT (mg)	Stirring rate (rpm)	Stirring time (min)	Sonication	Appearance
F1-IMT	25	25	25	–	–	25	1	10,000	15	500 W and 20 kHz	Clear
F2-IMT	33.33	33.33	–	33.33	11.11	–	1	10,000	5	500 W and 20 kHz	Clear
F3-IMT	28.57	28.57	–	37.5	12.5	–	1	10,000	5	500 W and 20 kHz	Clear
Formulations	Compritol 888 (%)	Oleic acid (%)	Tween 80 (%)	Lipoid S 75 (%)	Water:acetone:ethanol (5:2.5:2.5 v/v) %	Water (%)	IMT (mg)	Stirring rate (rpm)	Stirring time (min)	Sonication	Appearance
F4-IMT	30	25	10	25	10	–	1	10,000	5	500 W and 20 kHz	Foam formation
F5-IMT	33.33	33.33	–	22.22	11.11	–	1	10,000	5	500 W and 20 kHz	Phase separation
F6-IMT	20	20	–	37.63	12.37	10	1	10,000	5	500 W and 20 kHz	Foam formation
F7-IMT	28.57	28.57	–	28.57	14.28	–	1	10,000	5	500 W and 20 kHz	Foam formation

### Characterization and stability of NLCS containing IMT

The stability results of F1-IMT, F2-IMT, and F3-IMT are shown in Table 4. The particle sizes of F1-IMT, F2-IMT, and F3-IMT were found to be in accordance with desired value for NLCSs (below 100 nm) (Pandey & Khuller, 2005). PDI values did not exceed 0.5 for the formulations and they presented a homogenous distribution during the storage period. In addition, all results demonstrated that statistically significant differences were not observed. As a result, they ensured their physicochemical stability for up to 12 months.

**Table 4.** Stability of NLCSs formulations considering particle size, PDI, zeta potential, EE, and LC for 0 and 12 months’ storage at 5 °C, 25 °C (RH 60%), 40 °C (RH 75%) (*p*<.05).

Formulations	0 month					12 months				
	Particle size (nm ± SD)	PDI	Zeta Potential (mV ± SD)	EE (µg ± SD)	LC (%±SD)	Particle size (nm ± SD)	PDI	Zeta Potential (mV ± SD)	EE (µg ± SD)	LC (%±SD)
5 °C										
F1-IMT	86.93 ± 2.73	0.37 ± 0.14	–38.13 ± 2.16	951.74 ± 3.36	95.96 ± 1.8	96.27 ± 5.38	0.29 ± 0.09	–27.97 ± 3.14	949.78 ± 26.13	91.67 ± 2.50
F2-IMT	94.43 ± 2.28	0.35 ± 0.12	–37.9 ± 2.91	851.59 ± 4.73	86.31 ± 3.08	94.82 ± 5.27	0.23 ± 0.11	–26.77 ± 1.22	876.82 ± 25.10	87.26 ± 3.36
F3-IMT	82.43 ± 1.26	0.34 ± 0.21	–37.66 ± 2.66	962.49 ± 25.48	96.42 ± 1.48	95.96 ± 4.31	0.38 ± 0.05	–24.83 ± 4.11	947.9 ± 17.97	90.66 ± 0.62
25 °C (RH 60%)										
F1-IMT	86.93 ± 2.73	0.37 ± 0.14	–38.13 ± 2.16	951.74 ± 3.36	95.96 ± 1.8	96.26 ± 5.4	0.38 ± 0.14	–32.25 ± 2.9	941.75 ± 27.29	93.3 ± 1.14
F2-IMT	94.43 ± 2.28	0.35 ± 0.12	–37.9 ± 2.91	851.59 ± 4.73	86.31 ± 3.08	95.30 ± 4.95	0.35 ± 0.24	–29.76 ± 0.62	859.26 ± 18.63	85.343 ± 4.53
F3-IMT	82.43 ± 1.26	0.34 ± 0.21	–37.66 ± 2.66	962.49 ± 25.48	96.42 ± 1.48	91.77 ± 2.53	0.4 ± 0.12	–35.06 ± 5.01	948.82 ± 27.57	92.93 ± 2.59
40 °C (RH 75%)										
F1-IMT	86.93 ± 2.73	0.37 ± 0.14	–38.13 ± 2.16	951.74 ± 3.36	95.96 ± 1.8	93.38 ± 2.75	0.35 ± 0.13	–30.16 ± 0.24	944.52 ± 30.90	94.89 ± 3.76
F2-IMT	94.43 ± 2.28	0.35 ± 0.12	–37.9 ± 2.91	851.59 ± 4.73	86.31 ± 3.08	97.43 ± 3.66	0.22 ± 0.05	–29.23 ± 3.50	855.15 ± 30.93	86.07 ± 3.68
F3-IMT	82.43 ± 1.26	0.34 ± 0.21	–37.66 ± 2.66	962.49 ± 25.48	96.42 ± 1.48	93.23 ± 6.55	0.48 ± 0.05	–27.88 ± 1.11	949.66 ± 21.58	92.23 ± 2.54

### *In vitro* cytotoxicity studies

**Figure 1.**
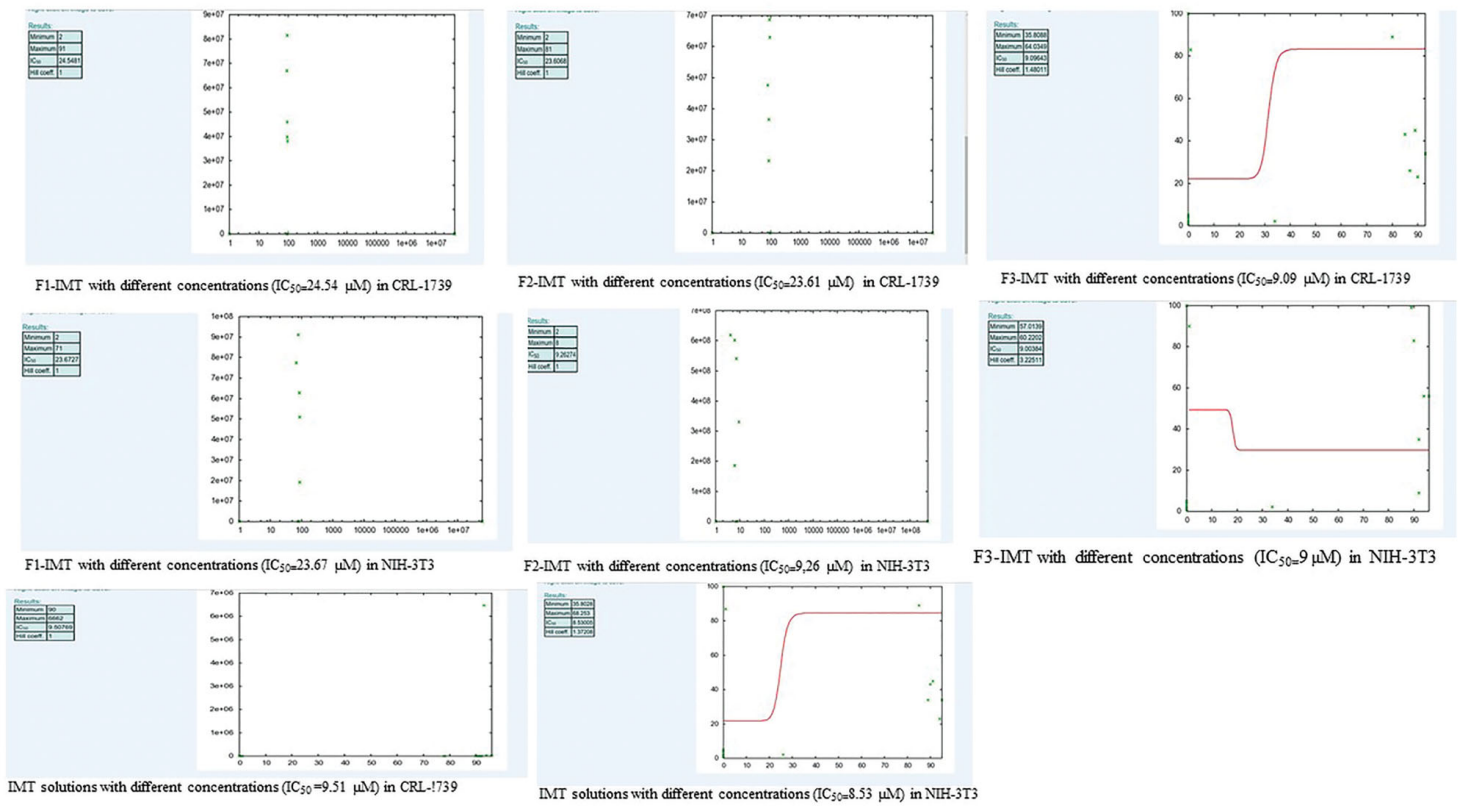
IC_50_ values of formulations.

The *in vitro* cytotoxicity studies of IMT solution, F1-IMT, F2-IMT, and F3-IMT were performed with NIH-3T3 and CRL-1739 cells. The IC_50_ values were found to be between 8.53 ± 2.7 and 24.54 ± 1.44 µM for NIH-3T3 and CRL-1739 cells (Figure 1). These values have shown that the administration of IMT solution and formulations with below IC_50_ concentrations to NIH-3T3 and CRL-1739 cells were not likely to cause toxicity and future administration of them with these concentrations can be considered as safely drug delivery systems.

### Preparation of [^99m^Tc]Tc-NLCS-IMT Formulations

**Figure 2.**
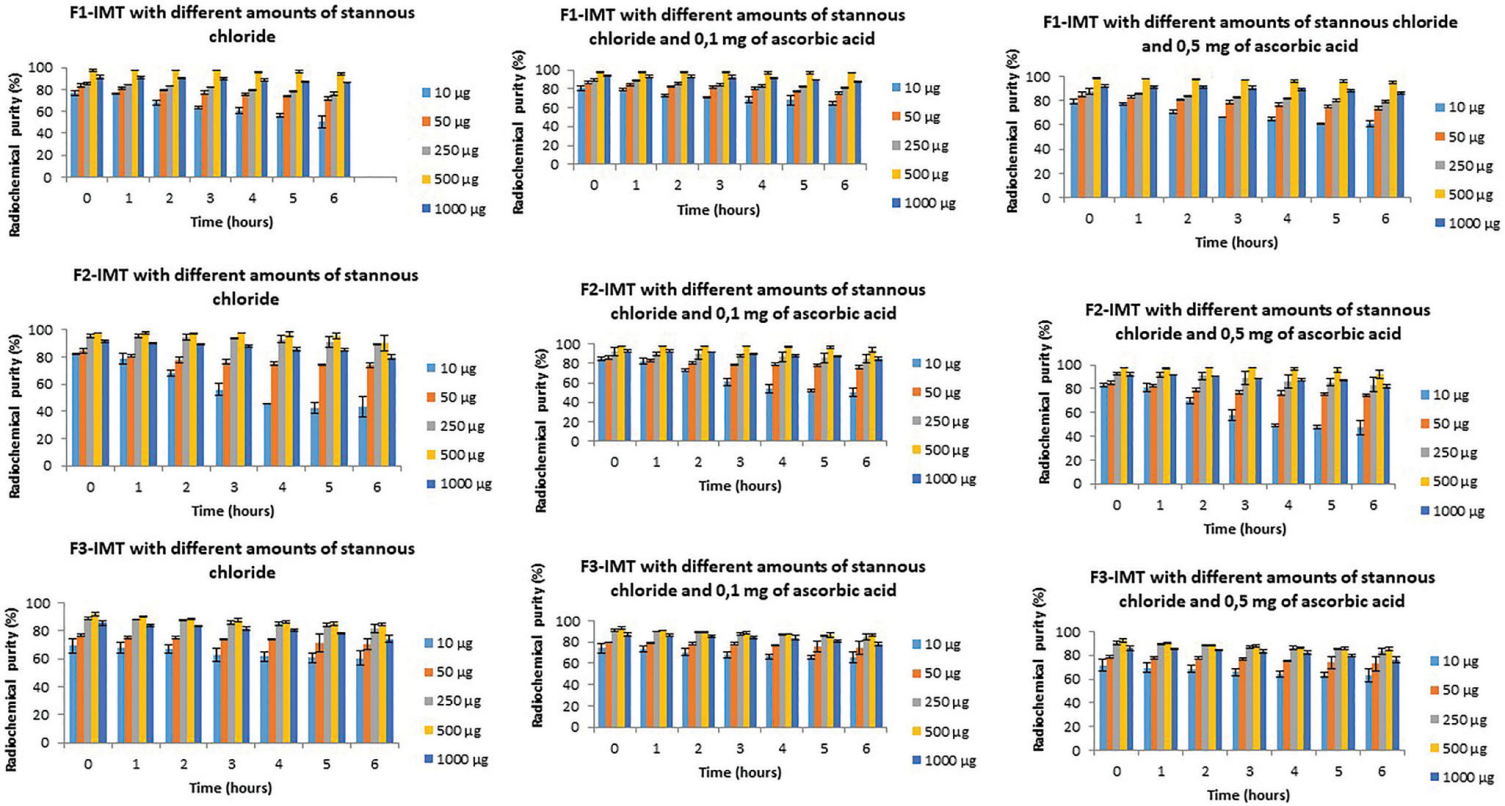
Radiochemical purity of NLCS-IMT formulations under different amounts of stannous chloride and ascorbic acid.

The formulations (F1-IMT, F2-IMT, and F3-IMT) were incubated with 1 mCi/0.1 mL of [^99m^Tc]Tc during 15 min. The radiochemical purity under different reducing and antioxidant agent’s amounts is shown in Figure 2. The RTLC chromatography showed retention factor (Rf)=0 corresponding to the [^99m^Tc]Tc-NLCS-IMT formulations and Rf = 1 corresponding to free Tc-99m when eluted using saline and pyridine:acetic acid:water (3:5:1.4). While preparation of [^99m^Tc]Tc-F1-IMT and [^99m^Tc]Tc-F2-IMT was successfully performed above 90% of radiochemical purity, [^99m^Tc]Tc-F3-IMT was obtained approximately 80% of radiochemical purity during 6 h at room temperature (*p*<.05). Furthermore, the ideal reducing and antioxidant agent’s amounts were found to be 500 µg of stannous chloride and 0.1 mg of ascorbic acid for all formulations.

### *In vitro* stability of [^99m^Tc]Tc-NLCS-IMT formulations

**Figure 3.**
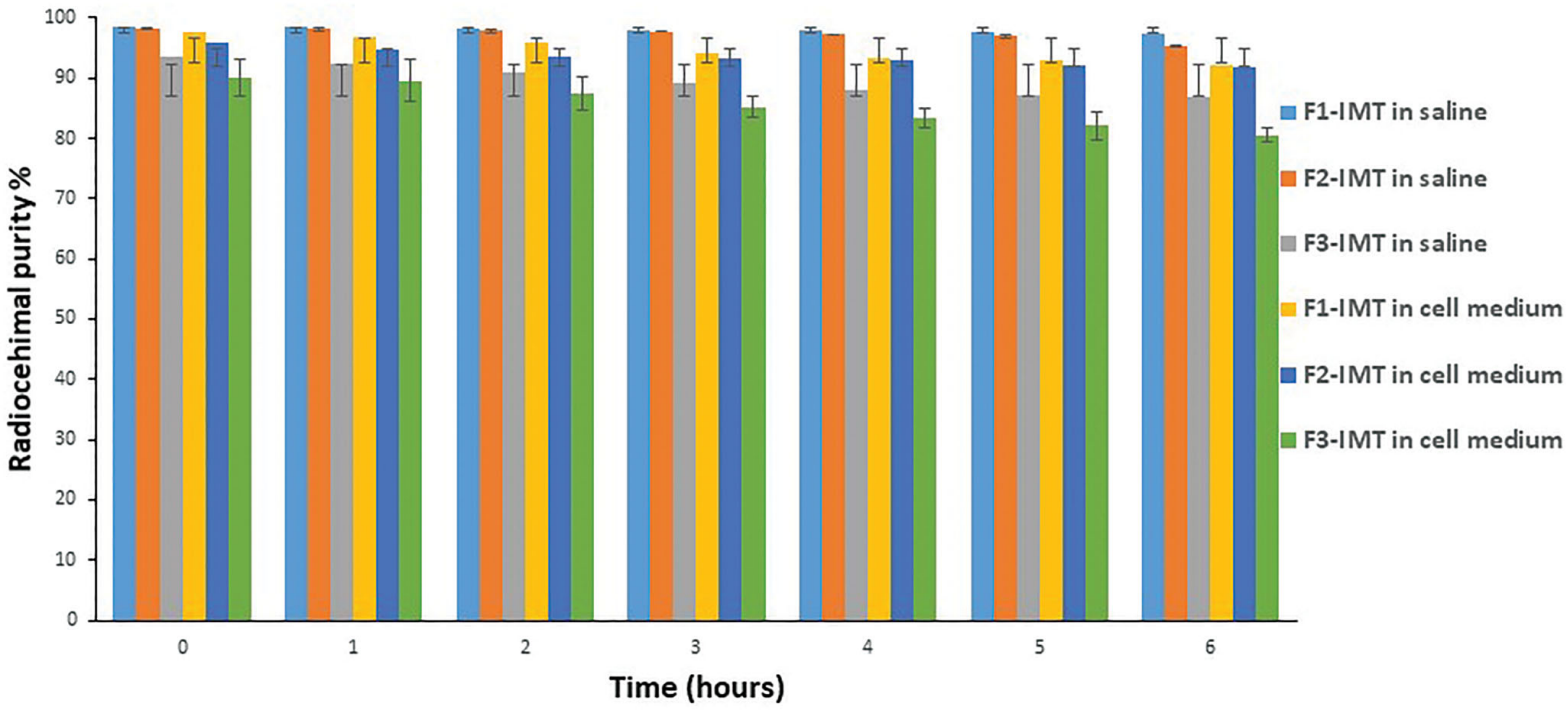
Radiochemical purity % of NLCS-IMT formulations in saline and cell medium.

The radiochemical purity (%) versus time of [^99m^Tc]Tc-NLCS-IMT formulations in saline and cell medium is shown in Figure 3. While radiochemical purity of [^99m^Tc]Tc-NLCS-IMT formulations was found to be between 80.56 ± 1.24 and 92.06 ± 0.98% in cell medium, the radiochemical purity of [^99m^Tc]Tc-NLCS-IMT formulations was found to be between 86.73 ± 0.54 and 97.41 ± 0.14% in saline for 6 h (*p*>.05) (Figure 3).

The radiochemical purity of F1-IMT and F2-IMT did not decrease below 90% and these formulations were more stable in saline, while the radiochemical purity of F3-IMT was found to be between 86% and 80% in cell medium and saline. The basic principle in the preparation of radiolabeled systems is to achieve high radiochemical purity (over 90%) and stability as possible. It has also been found that radiolabeled F1-IMT and F2-IMT formulations have more than 90% of radiochemical purity during radiolabeling studies. The results showed that it would be more appropriate to use these two formulations in cell binding studies.

### Cell binding studies with [^99m^Tc]Tc-NLCS-IMT formulations

The cell binding ratio of [^99m^Tc]NaTcO_4_, [^99m^Tc]Tc-F1-IMT, and [^99m^Tc]Tc-F2-IMT and targeting/non-targeting cell uptake ratio is shown in Table 5. While the cell binding ratio reached to peak value for [^99m^Tc]Tc-F1-IMT and [^99m^Tc]Tc-F2-IMT at 60 min, it has decreased at 120 min. Also the cell binding ratio of [^99m^Tc]Tc-F1-IMT and [^99m^Tc]Tc-F2-IMT to CRL-1739 and NIH-3T3 was found to be different (*p*<.05) and maintained at a higher rate in CRL-1739 (Table 5). [^99m^Tc] NaTcO_4_ was found to have the lowest binding ratio.

**Table 5.** Cell binding and targeting/non-targeting ratio of [^99m^Tc] NaTcO_4_, [^99m^Tc]Tc-F1-IMT, and [^99m^Tc]Tc-F2-IMT.

Time (min)	[^99m^Tc] NaTcO_4_		[^99m^Tc]Tc-F1-IMT		[^99m^Tc]Tc-F2-IMT		[^99m^Tc] NaTcO_4_	[^99m^Tc] Tc-F1-IMT	[^99m^Tc] Tc-F2-IMT
	Cell binding ratio of NIH-3T3	Cell binding ratio of CRL-1739	Cell binding ratio of NIH-3T3	Cell binding ratio of CRL-1739	Cell binding ratio of NIH-3T3	Cell binding ratio of CRL-1739	Targeting/ non-targeting ratio	Targeting/ non-targeting ratio	Targeting/ non-targeting ratio
30	15.13 ± 4.48	17.22 ± 7.56	21.31 ± 6.34	28.78 ± 8.61	12.63 ± 1.63	30.69 ± 9.21	1.14 ± 0.09	1.35 ± 0.22	2.42 ± 0.89
60	20.56 ± 9.03	24.89 ± 3.42	25.02 ± 8.3	50.99 ± 8.16	28.28 ± 7.91	39.41 ± 7.34	1.21 ± 0.18	2.03 ± 0.31	1.39 ± 0.56
120	18.34 ± 3.12	15.13 ± 4.27	24.68 ± 4.67	25.08 ± 2.94	26.41 ± 5.03	31.17 ± 6.18	0.83 ± 0.11	1.01 ± 0.9	1.18 ± 0.44

The high binding ratio of radiolabeled molecules/systems in the target tissue allows us to obtain good quality images, while also reducing radiation damage in the non-target tissue. For this reason, it is desired that the radiolabeled formulations have 2 or above values for targeting/non-targeting cell uptake ratio (Fazlil et al., 2012). While high targeting/non-targeting cell uptake ratio was found to be 2.03 ± 0.31 for F1-IMT formulation at the 60 min and 2.42 ± 0.89 for F2-IMT formulation at the 30 min, it was found to be below 2 for [^99m^Tc] NaTcO_4_ at each time (Table 5).

## Discussion

In this study, IMT was chosen as the active ingredient. IMT has specific activity on Bcr-Abl, PDGFR, and c-Kit and effectively used in the treatment of GIST (Deininger et al., 2005). However, it has some limits such as adverse effect, toxicity, extensive first pass metabolism, and rapid clearance in clinical administrations. These symptoms can be reduced by using a lower dose than therapeutic dose or using nano systems that can create a more effective treatment and provide targeting to the desired area (Caboi et al., 2005; Sawant & Torchilin, 2010; Negi et al., 2014). Based on these considerations, NLCSs containing IMT formulations were prepared. The amount of IMT used in conventional therapy is 400 mg/day (Qu et al., 2016). Herein, the amount of IMT was adjusted to 1 mg as lower IMT dose than the conventional therapeutic dose. In the light of this information, developed NLCSs containing IMT dose would greatly improve its potential chemotherapeutic effect with low dose.

Developed formulations were characterized in terms of particle size, zeta potential, PDI, EE, and LC values. The particle size of NLCSs plays an important role in drug delivery and affects targeting efficiency. It is well known that nano systems with various particle sizes have the different distribution in the organs (Almeida et al., 2011; Hoshyar et al., 2016). For example, the nano systems with diameters larger than 100 nm will activate the complement system and be quickly removed from the blood stream, accumulating in the liver and spleen. Nano systems around 20–50 nm could increase the accumulation in the bone marrow. However, it is still unclear how the particle size affects the targeting effect. Herein, new NLCSs with particle sizes below 100 nm were obtained (Table 4). These particles will avoid the clearance of IMT by the reticuloendothelial system and provide the carrier system containing drug to stay in circulation (Selvamuthukumar & Velmurugan, 2012).

When PDI or particle size distribution interval is small, the particles have similar sizes and the system is considered to be homogeneous in this aspect. Generally, PDI values below 0.5 are reported to be acceptable (Badilli et al., 2015). In our study, PDI value below 0.5 was chosen to guarantee that the sizes of developed formulations were especially homogeneous (Table 4).

The zeta potential indicates the net electrostatic load on the particle surface and is an important parameter in the evaluation of stability of colloidal systems. Also, it effects the interaction of formulation with biological system. The usage of high surfactant concentration in the preparation process leads to high zeta potential value and also reduction in particle size (Heurtault et al., 2003; Honary & Zahir, 2013; Tefas et al., 2015). Therefore, we decided that zeta potential values of developed formulations are acceptable with an interval of 0 mV–(−30) mV and support low particle size of formulations (Table 4).

Encapsulation of hydrophilic drugs into the lipophilic matrices of lipid nano carrier systems is difficult. When some hydrophilic drugs loaded lipid nano formulations were prepared by using a variety of production methods and formulation parameters, encapsulation efficiency values were observed to be high (Singh et al., 2010; Yassin et al., 2010). In this study, high EE and LC values (Table 4) were obtained. Therefore, F1-IMT, F2-IMT, and F3-IMT were found to be proper for carrying the IMT in intravenous route thanks to their particle sizes, homogeneous particle size distribution, zeta potential, EE, and LC values.

NLCSs as a delivery vehicle need to exert their efficacy under different storage conditions and should not be subjected to dramatic changes. Therefore, studying the stability of NLCSs are of great significance for its administration and indicate the physicochemical characteristics of formulations (Bunjes, 2010). As shown in Table 4, particle sizes, zeta potentials, and PDI values of the formulations are different from each other (*p*<.05), but these values of each formulation remained the same (*p*>.05) during storage conditions and time intervals. According to these results, all NLCS formulations were found to be stable at even high temperature and humidity for 12 months.

Cytotoxicity studies using MTT assay were performed to evaluate IC_50 of_ IMT solution, F1-IMT, F2-IMT, F3-IMT on the NIH-3T and CRL-1739 cells. IC_50_ value has important role to determine the cytotoxic dose of drug in drug delivery systems. Results in Figure 1 showed that F1-IMT, F2-IMT, and F3-IMT have an IC_50_ value of between 23.61 and 9.26 μM for NIH-3T and CRL-1739 cells. According to these results, the formulations can be applied safely in NIH-3T and CRL-1739 cells below IC_50_ value that is obtained from cytotoxicity studies.

[^99m^Tc]Tc is an ideal radionuclide to radiolabel new drug delivery systems. The reducing agent is crucial for radiolabeling studies with [^99m^Tc]Tc. The colloid structure occurs in the radiolabeled system and radiochemical purity begins to decrease by adding reducing agent in high concentrations. In the presence of lower concentrations of reducing agent, free technetium ratio decreases. In both cases, radiochemical purity of radiolabeled system is adversely affected. Mostly stannous salts are preferred to use as reducing agents in radiolabeling process (Liu, 2005; Tsionou et al., 2017). In this study, stannous chloride was used as reducing agent. The effect of different reducing agent concentrations was evaluated and optimum stannous chloride amount was found to be 500 μg for radiolabeling (Figure 2). [^99m^Tc]Tc radiolabeled systems may have auto radiolysis during preparation, release, and storage. It will decrease the stability and targeting capability of formulation. Therefore, it is very important to use stabilizer to minimize the auto radiolysis. The stabilizers are often antioxidants, such as ascorbic acid, gentisic acid, and p-aminobenzoic acid (Liu, 2005). Herein, ascorbic acid was used as an antioxidant agent. The effect of antioxidant agent concentration on the radiochemical purity was evaluated, and optimum ascorbic acid amount was found to be 0.1 mg for all formulations (Figure 2).

The poor targeting/non targeting cell uptake ratio causes stability problems. In this study, the stability of [^99m^Tc]Tc-F1-IMT and [^99m^Tc]Tc-F2-IMT was found to be higher than [^99m^Tc]Tc-F3-IMT in saline and cell medium during 6 h (Figure 3) because of this, cell binding studies were performed with [^99m^Tc]Tc-F1-IMT and [^99m^Tc]Tc-F2-IMT.

A high targeting/non targeting cell uptake ratio of radiolabeled systems is critical in clinical administrations. It can affect the quality of target organ images and be localized in the nontargeting organs and cause injury in these tissues (Maruvada et al., 2005). According to *in vitro* cell binding studies, [^99m^Tc]Tc-F1-IMT and [^99m^Tc]Tc-F2-IMT showed higher cell binding ratio on CRL-1739 than NIH-3T3 when compared to [^99m^Tc] NaTcO_4_ and the highest cell binding ratio was observed with [^99m^Tc]Tc-F1-IMT for CRL-1739 cells at 60 min (Table 5). Also, the cell binding ratio has been changed at time intervals. Despite the exact reason not being explained, these changes may be due to the pharmaceutical and radionuclide parts of radiolabeled system, affinity of radiolabeled formulations to cells, and time.

## Conclusions

In this study, IMT-NLCS formulations (F1-IMT, F2-IMT, and F3-IMT) were prepared and their targeting efficiency was evaluated on NIH-3T3 as fibroblast cells and CRL-1739 as gastric adenocarcinoma cells. After the preparation, NLCSs formulations exhibited proper characterization profiles with their nanosize, zeta potential values and effective encapsulation efficiency and LC. In addition, a simple and direct radiolabeling process was performed and optimized in terms of reducing agent and stability in different media to evaluate of cellular binding capacity of NLCSs.

In conclusion, stable IMT-NLCSs formulations were prepared. ^99m^Tc-radiolabeled formulations exhibited promising results depending on not only physicochemical characterization properties but also *in vitro* cellular binding profile. It has been seen that the combination of IMT, NLCS, and [^99m^Tc] Tc may contribute to targeting of IMT on gastric adenocarcinoma cells.
